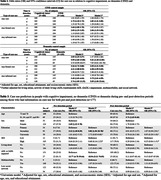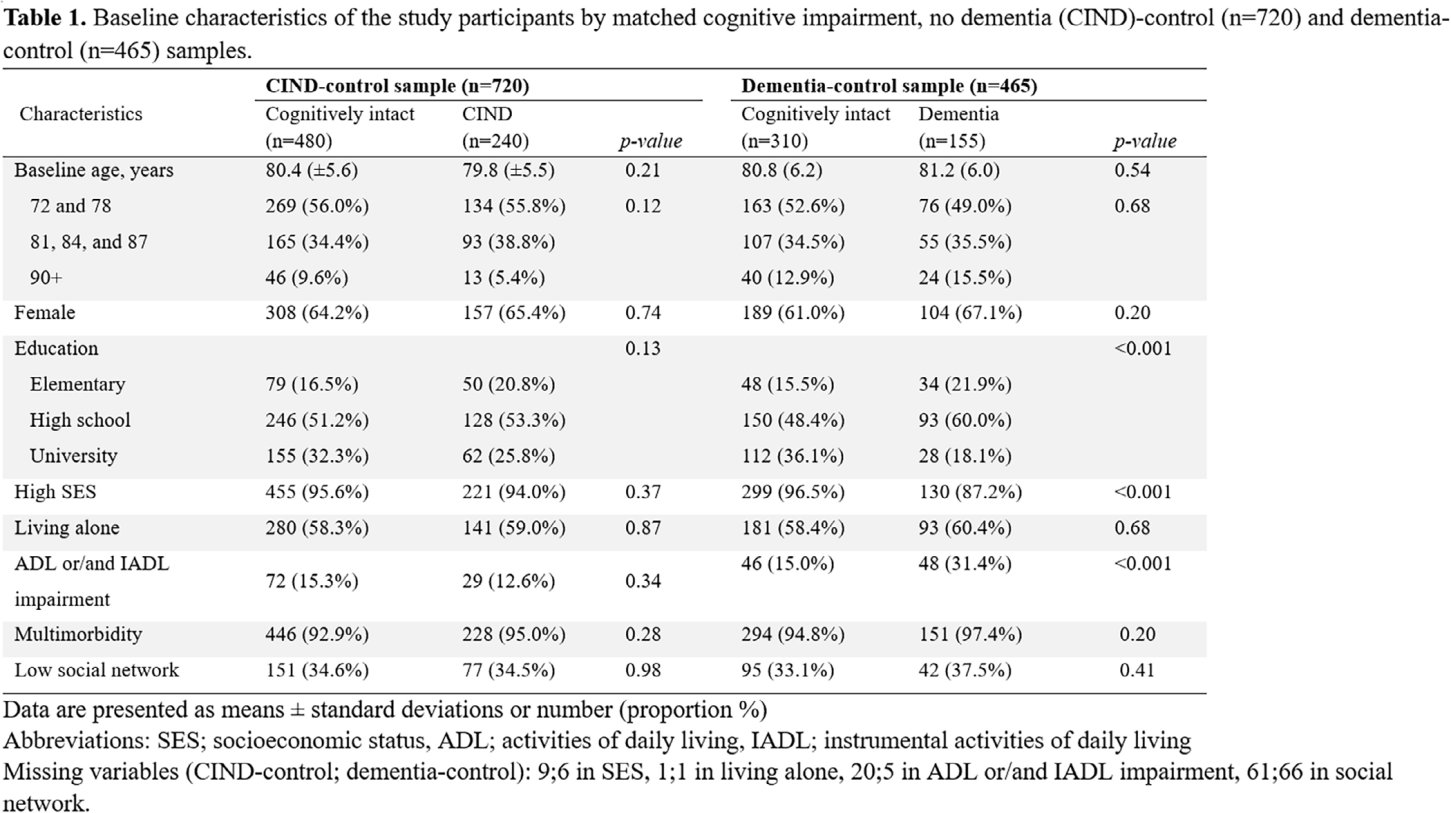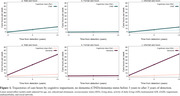# Formal and informal care use before, during, and after detection of cognitive disorders: a population‐based longitudinal study

**DOI:** 10.1002/alz.085931

**Published:** 2025-01-09

**Authors:** Sakura Sakakibara, Abigail Dove, Jie Guo, Giulia Grande, Britt‐Marie Sjölund, Janne Agerholm, Laura Fratiglioni, Weili Xu

**Affiliations:** ^1^ Aging Research Center, Karolinska Institutet, Stockholm, Stockholm Sweden; ^2^ Aging Research Center, Karolinska Institutet, Stockholm Sweden; ^3^ China Agricultural University, Beijing, Beijing China; ^4^ Aging Research Center and Center for Alzheimer Research, Karolinska Institutet, Stockholm Sweden; ^5^ University of Gävle, Gävle, Gästrikland Sweden; ^6^ Aging Research Center, Karolinska Institutet, Solna, Stockholm Sweden; ^7^ Aging Research Center and Center for Alzheimer Research, Karolinska Institutet‐Stockholm University, Stockholm Sweden; ^8^ Center for International Collaborative Research on Environment, Nutrition, and Public Health, Tianjin China

## Abstract

**Background:**

Dementia is strongly linked to increased care use, but the use of formal and informal care throughout dementia journey remains unclear.

**Method:**

Within a population‐based cohort study, we identified 240 older adults (aged ≥78 years) with who developed CIND and 155 with incident dementia. These participants were matched to 480 and 310 cognitively intact participants, respectively, and their formal and informal care use and care hours were compared with a control groups before and after diagnosis of cognitive disorders.

**Result:**

Compared to cognitively intact participants, those with CIND were more likely to use formal care at the detection (odds ratio [OR] 2.06, 95% confidence interval [95%CI] 1.19‐3.58). Compared to cognitively intact participants, those with dementia were more likely to use formal care before (OR 2.55, 95% CI 1.21‐5.39) and at the wave of (OR 4.38, 95% CI 1.99‐9.65) diagnosis, while informal care use was greater at the wave of (OR 3.75, 95% CI 2.01‐6.99) and after diagnosis (OR 5.05, 95% CI 1.00‐25.64). In linear regression analysis, CIND and dementia were related to a faster increase in informal care hours (β: 5.52, 95% CI 2.87, 8.17/ β: 30.26, 95% CI 24.40, 36.12) compared to controls. Among individuals with CIND/dementia, older age, female sex, secondary educational attainment, and impairments in activities of daily living (ADL) at baseline were significantly associated with formal/informal care use.

**Conclusion:**

Compared to cognitively intact individuals, those with CIND were associated with increased formal care use at detection, while people with dementia were associated with increased use of formal care already before diagnosis and informal care from diagnosis. CIND and dementia are associated with increased informal care hours. Age, sex, education, and ADL may predict greater care use among people with CIND/dementia.